# The BioGenome Portal: a web-based platform for biodiversity genomics data management

**DOI:** 10.1093/nargab/lqaf020

**Published:** 2025-03-22

**Authors:** Emilio Righi, Roderic Guigó

**Affiliations:** Centre for Genomic Regulation (CRG), The Barcelona Institute for Science and Technology, C/ Dr. Aiguader 88, Barcelona 08003 Catalonia, Spain; Centre for Genomic Regulation (CRG), The Barcelona Institute for Science and Technology, C/ Dr. Aiguader 88, Barcelona 08003 Catalonia, Spain; Departament of Medicine and Life Sciences, Universitat Pompeu Fabra (UPF), C/ Dr. Aiguader 80, Barcelona 08003, Catalonia, Spain

## Abstract

Biodiversity genomics projects are underway with the aim of sequencing the genomes of all eukaryotic species on Earth. Here we describe the BioGenome Portal, a web-based application to facilitate organization and access to the data produced by biodiversity genomics projects. The portal integrates user-generated data with data deposited in public repositories. The portal generates sequence status reports that can be eventually ingested by designated metadata tracking systems, facilitating the coordination task of these systems. The portal is open-source and fully customizable. It can be deployed at any site with minimum effort, contributing to the democratization of biodiversity genomics projects. We illustrate the features of the BioGenome Portal through a number of specific instances. One such instance is being used as the reference portal for the Catalan Initiative for the Earth Biogenome Project, a regional project aiming to sequencing the genomes of the species of the Catalan linguistic area.

## Introduction

Biodiversity genomics projects are underway that aim to sequence the genomes of the >1.2 million known eukaryotic species on Earth [1]. The impact of these projects will be unprecedented as it will radically alter our understanding of life on Earth. These projects differ in size and scope: some target geographic locations, such as the Darwin Tree of Life (DTOL) [2], the Catalan Initiative for the Earth Biogenome Project (CBP) [3], the European Reference Genome Atlas (ERGA) [4], or the California Conservation Genomics Project [5], others target specific taxa, such as the vertebrate genome project (VGP) [6] or the Bird 10 000 Genomes [7]. They also differ in funding schemas, organization, and structure. The Earth Biogenome Project (EBP) [8] acts as a network umbrella to all these projects, providing common guidelines and standards (operational, but also ethical and legal) for sample collection and processing, sequencing and assembling, annotation, data analysis, IT, and informatics. As of 2024, the EBP includes 58 affiliated projects worldwide. Many of these projects are networks themselves, composed of multiple nodes (for instance, each European country is a node of ERGA, which includes, in addition, some regional nodes). The EBP can thus be properly described as a network of networks.

These projects (or even the nodes within a project) are operationally independent and carry out all of the steps of the genome sequencing process autonomously: from the collection of the biological samples to the production of the genome assembly. The EBP uses Genomes on a Tree (GoaT) [9] for coordination. GoaT is a centralized resource sponsored by Tree of Life programme (https://www.sanger.ac.uk/programme/tree-of-life/) that collates observed and estimated genome-relevant metadata—including genome sizes and karyotypes—for eukaryotic species, and that also holds declarations of current and planned activity across the EBP nodes. GoaT is also the official sequence status tracker of the EBP.

Here we present the BioGenome Portal (BGP), an easy to use web-based application designed to manage, integrate, and track data generated by individual biodiversity genomics projects. In addition to the data collected in centralized services, the BGP allows users to link multiple types of information, such as images, vernacular names, sample metadata, user-defined metadata, scientific publications, literature texts, etc, directly to the species. In addition, by generating GoaT-compliant sequencing status reports, the BGP could be used to facilitate GoaT keeping the sequencing status of the EBP up to date. The BGP, however, can be used, more generally, to provide genome information on any collection of species, such as botanical or zoological gardens, regardless of whether they are part of the EBP, and to disseminate data specific to a particular site. To illustrate the flexibility of the BGP, we have implemented instances corresponding to two EBP regional initiatives, the CBP and ERGA, to the entire EBP and to a lichens herbarium.

A Catalan version of the abstract of the article can be found at: https://doi.org/10.5281/zenodo.14060240.

## Materials and methods

### System architecture and configuration

**Figure 1.**
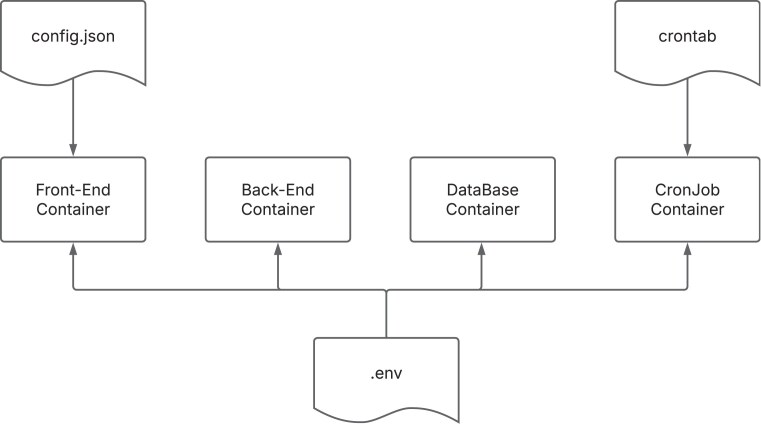
Schema depicting the configuration files injected in the BGP’s containers.

The BGP is composed of six Docker [10] containers orchestrated by Docker Compose. With the use of a docker-compose configuration file, it is possible to configure the application infrastructure and start all containers with a single command. This greatly simplifies application infrastructure management, improving application portability between development and production environments and facilitating continuous application integration and deployment. In addition, each container can be customized with individual configuration files. This flexibility allows for adaptation to the diverse needs of biodiversity research projects. Through these configuration files, the BGP can seamlessly import and process data from external sources and customize its charts to meet specific project requirements (Fig. 1).

*Back-end container*: The back-end container consists in a Python Flask application (https://flask.palletsprojects.com) that exposes an Application Programming Interface (API) via a uWSGI web server, that in turn interfaces with the NGINX [11] reverse proxy of the front-end container, to perform CRUD (create, read, update, and delete) operations against the database container. The .env file, primarily utilized by the back-end container, contains essential environment variables. These variables include admin credentials for authorized access, taxonomic identifiers for phylogenetic trees in the User-Interface (UI), International Nucleotide Sequence Database Collaboration (INSDC) Bioproject accession for assembly retrieval, a comma-separated list of project names for fetching sample metadata from the EBI Biosamples API, and other configuration variables crucial for inter-container communication within the Docker environment.

*Front-end* *container*: The front-end container consists of a Vue.js (https://vuejs.org/) single-page application (SPA) served via a NGINX reverse proxy. This container is accessible via a web browser and communicates with the back-end container via API. The SPA is composed of a content management system (CMS) section where authenticated users are able to manage the data present in the BGP database, and an open access section that contains many cutting-edge features to enhance data visualization. A set of JavaScript Object Notation (JSON) configuration files are used to configure the front-end container. These files define various elements of the user interface, ensuring consistency and ease of modification across different sections of the portal.

A general JSON configuration file is used to configure the general settings of the UI, such as:

The title and logo of the UI; the boolean flags to enable the CMS, the GoaT sequencing feature and the EBP related metrics filters; the path to a tracking code script; a languages array to specify the supported languages with their respective codes and names; a Wikipedia section to provide the URLs to Wikipedia in each supported language.

The other JSON files are used to customize, respectively, the pages to display, the filters to query the database, the columns of the data tables, and the charts to be displayed in each page.

These JSON files serve as a centralized configuration source, ensuring the web application is easily customizable and maintainable. By defining UI elements, charts, filters, and metadata in a structured format, it allows for a dynamic and multilingual user experience.

*Cronjob container*: The cronjob container schedules authenticated requests to the API, triggering specific tasks for data import and updates in the database. The crontab file, residing within the cronjob container, configures scheduled jobs, providing precise control over automated data management processes.

*Database container*: The database container is composed of a MongoDB (https://www.mongodb.com) Docker image where all the data are stored. MongoDB uses a flexible document data model: data in the database can be stored without having to define a strict schema beforehand. In MongoDB, data are stored as documents, which are similar to JSON objects and can be easily modified and updated. One of the advantages of this flexible schema is that it allows for easy modification and expansion of the data model. As new requirements arise, users can easily add new fields to their documents without the need for complex schema migrations or downtime.

*Celery and* *Redis* *containers*: To manage and execute scheduled background jobs, we utilized a combination of Redis (https://redis.io) and Celery (https://docs.celeryq.dev) containers. Redis, an in-memory data structure store, served as the message broker, facilitating communication between different components of the system. Celery, a distributed task queue, was employed to schedule and run tasks asynchronously, ensuring efficient handling of background processes. This setup enabled the system to handle periodic tasks reliably and efficiently, with Redis ensuring quick message passing and Celery managing the execution of scheduled jobs.

### Data import and export

The BGP offers robust data management features, providing researchers a seamless way to work with biodiversity genomic data. The BGP is API-centered thus, importing and exporting data can also be done by directly querying the API.

*Data export:*All the organisms-related data can be exported either in JSON, JavaScript Object Notation Lines (JSONL), or tab-separated values (TSV) format, allowing users to generate detailed reports of the data contained in the database.

**Figure 2.**
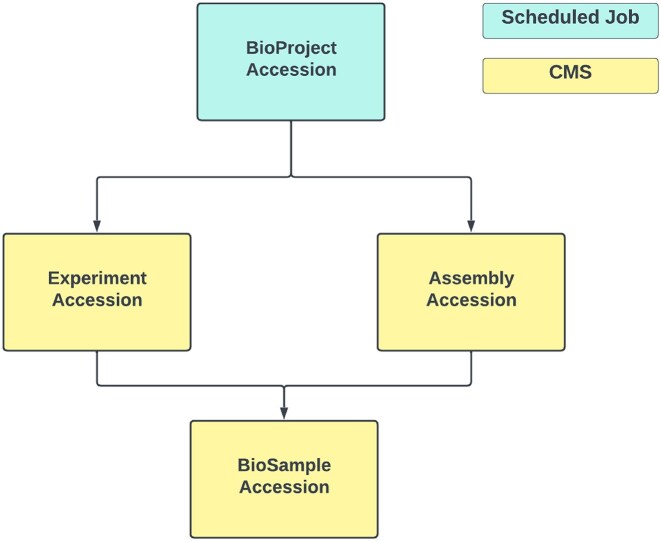
Schema depicting the INSDC data import strategies of the BGP. Via scheduled job all the INSDC data under a specific bioproject accession can be imported. Via CMS individual INSDC data can be imported via their accession.

*INSDC* *data import*: The INSDC related data can be imported into the BGP database either via cronjob or CMS (Fig. 2). Through a cronjob process, assembly metadata associated with the specified BioProject accession number declared in the .env file is systematically retrieved from NCBI Datasets [12] and stored in the database. This mechanism ensures comprehensive data tracking for the specified BioProject. For each imported assembly, the system seamlessly incorporates the associated BioSample metadata and, if available, the associated experiment metadata. In addition, the system performs checks to identify any BioSamples derived from those associated with the assembly and imports them along with their associated experiments.

Alternatively, authenticated users can use the CMS feature to import experiments, BioSamples, and assemblies individually by specifying their respective accession numbers.

Another cronjob functionality allows direct import of BioSample metadata from EBI BioSamples. This process retrieves all BioSamples that match the project name, declared in the .env file, specified in their metadata. For each imported BioSample, the system verifies the presence of coordinates and a country name in its metadata, ensuring data integrity and accuracy.

*Samples* *metadata import*:Spreadsheets containing sample metadata can be uploaded into the database via CMS. The only spreadsheet’s columns required are the unique identifier of the sample, the scientific name and the taxonomic identifier, the remaining columns will be stored as metadata. The system will also try to parse any coordinates if columns containing latitude and longitude values are present in the spreadsheet.

*GoaT* *sequencing status*: The BGP offers a powerful feature that allows users to upload or download detailed TSV files containing the sequencing status of all organisms stored in the database. This functionality is designed to provide researchers with a convenient means of aggregating and sharing important sequencing status information. The TSV file format conforms to the established GoaT sequencing report template. This alignment with the GoaT template ensures that the exported data are standardized, making it easily compatible with existing genomic data management systems and facilitating seamless integration with broader genomics research initiatives. Researchers can rely on these TSV files to generate comprehensive sequencing status reports for their data, ensuring data consistency and facilitating collaboration within the biodiversity genomics community. The sequencing status of each species can be updated either automatically or manually (in case of pre-INSDC submission).

*Genome annotations*: BGP offers the capability to enrich genome assemblies by incorporating genome annotations. These annotations, available in gzipped and tabindex formats, can be uploaded, via CMS, for integration with existing genome assemblies.

Users can upload annotation files through two distinct methods:

Users can directly upload annotation files to the portal’s server. If a volume is mounted within the server container, the path to this volume can be declared in the environment (.env) file. This enables users to upload annotation files stored locally on their systems directly to the portal for integration with genome assemblies.

Alternatively, users can choose to host annotation files on external servers, such as Amazon S3. In such cases, users can provide links to the annotation files during the upload process. The portal will then access and integrate these annotation files into the genome assemblies as needed.

Additionally, users have the option to upload a chromosome aliases text file. This file facilitates the mapping of sequence names within the annotation files to corresponding chromosome names in the genome assemblies, ensuring precise integration of annotation data.

These annotations can be visualized within the portal’s genome browser (see UI components).

### Server specifications

The instances of the BGP are currently hosted on two different servers.

*CBP instance*: The CBP instance is currently hosted on a AWS EC2 t3a.medium instance, with an AMD EPYC 7000 series processor with two vCPUs, 4 GB of RAM, and 10 GB of general purpose SSD storage. The t3a.medium instance is a burstable performance instance, meaning it provides a baseline level of CPU performance with the ability to burst above the baseline when needed, using CPU credits. This instance is part of the T3 series, which are cost-effective and ideal for workloads that require a balance of compute, memory, and network resources, with the flexibility to handle occasional spikes in usage. This instance type is generally used for smaller workloads and applications that require moderate baseline performance with the capability to handle occasional bursts of activity.

*E* *BP*,*ERGA*, *andLichenoteca instances*: These instances are hosted on a virtual machine provided by the Centre for Genomic Regulation. The virtual machine is powered by an Intel Xeon E5-2620 processor, featuring four physical cores within a single socket.

The virtual machine is equipped with 4 GB of RAM and 4 GB of swap memory, which is adequate for handling the multiple simultaneous processes and database operations that the instances require.

Storage is managed through a Network File System (NFS), providing around 70 GB of space. The use of NFS offers several advantages in a research environment, such as centralized storage management, ease of backup and recovery, and the ability to scale storage as needed.

### UI components

The BGP has a variety of useful visualization tools such as charts, a hyperbolic tree (Supplementary Fig. S1), maps (Supplementary Figs S2 and S3), and an integrated genome browser (Supplementary Fig. S4). The charts in the BGP provide a visual representation of the data contained in the database, such as the INSDC submission status of the project, the submission trend of INSDC related data, the sequencing platform used, the genome assembly level and the habitat of the collected samples.

The hyperbolic tree (https://github.com/glouwa/d3-hypertree) allows users to explore the relationships between different organisms based on their taxonomy. By selecting a specific taxonomic parent, users can visualize all the related data under that specific taxon.

The BGP features an interactive 2D map (Leaflet.js, https://leafletjs.com/) that can be used to display the organism’s geographical distribution and the organisms collected by each country. With this feature, BGP makes it easy to browse and analyze the distribution of organisms in different geographic regions.

The integrated genome browser (JBrowse2) [13] provides users with the ability to explore and download FASTA sequences of chromosome-level genomes using a RefGet API-compliant plugin (https://github.com/guigolab/jbrowse-plugin-refget-api). This plugin retrieves the nucleotide sequences from servers that are compliant with RefGet API [14] standards, without the need to download the entire genome assembly. In addition, authorized users can associate genomic annotations with genomes in the CMS section of the portal. These annotations can be visualized alongside the corresponding genomes.

### Database model

**Figure 3.**
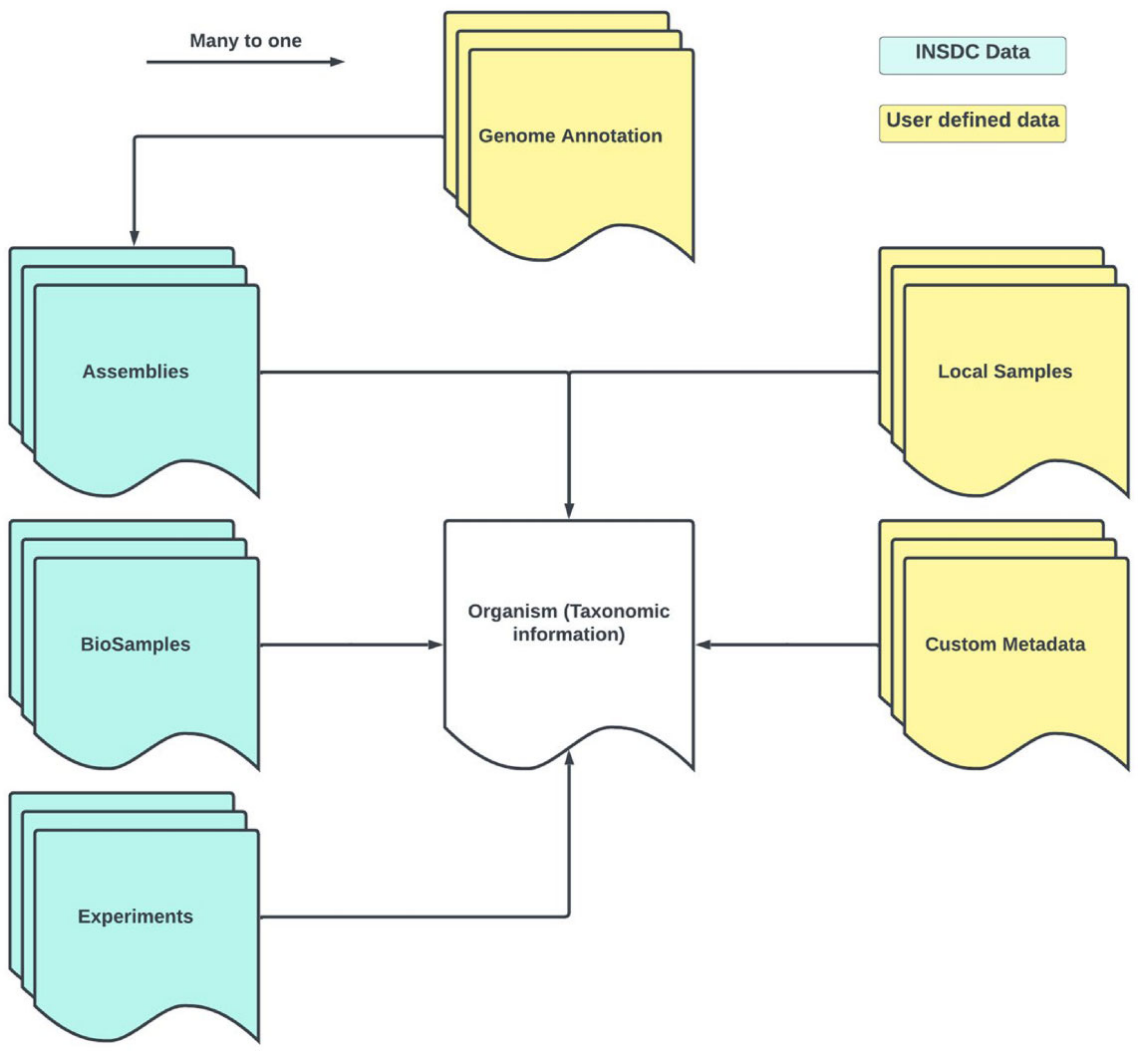
Schema depicting the organism-centered database model. Local Sample, Custom Metadata and Genome Annotation are the data that can be inserted by users. Assemblies, BioSamples and Experiments are the data that can be imported from INSDC via their accession number.

The BGP database model is organism-centered (Fig. 3). At the core of this model is the association of both locally curated data and data from the INSDC with a unified set of taxonomic details. There are several important advantages to this design approach. It promotes an intuitive and structured organization of data, allowing researchers to easily navigate and retrieve information about specific organisms. It also ensures the consistency and coherence of the data representation, eliminating redundancy and potential conflicts in taxonomic classifications. In addition, an organism-centered model simplifies the process of integrating external INSDC data with locally generated information to create a consistent and integrated repository. Ultimately, this design facilitates efficient data retrieval, comparative analysis, and cross-referencing between different datasets.

A detailed description on how to configure, run and deploy a BGP instance can be found in the README file of the github repository (https://github.com/guigolab/biogenome-portal, DOI: 10.5281/zenodo.8314305.)

## Results

We have developed the BGP (https://github.com/guigolab/biogenome-portal) to facilitate access to data produced by biodiversity genomics projects and interface public data repositories. The BGP is a web-based application that transparently brings together into a single virtual platform, data generated under a given biodiversity project, including both data already published on INSDC, as well as data declared prior to INSDC submission. In addition, through the BGP, it is possible to attach to the sequenced organisms information not available at the INSDC, such as photos, links to relevant scientific publications and other resources, historical notes, descriptions of sequenced species, species names in local languages, genome structural and functional annotations, and any other user-defined information data.

The technical details of the BGP architecture are described in “Materials and methods” section. The BGP repository can be found at https://github.com/guigolab/biogenome-portal, which also contains all the details to configure, run, and deploy it. Here we describe four use cases that demonstrate the adaptability, flexibility, and scalability of the BGP. The first use case is the BGP instance dedicated to the CBP [3]. The second case is the BGP instance corresponding to ERGA. These cases highlight the potentiality of the portal to supporting regional biodiversity genomics efforts. The third case corresponds to the entire EBP. This instance showcases the capacity of the portal to deal with large amounts of data. Finally, we have implemented an instance corresponding to a lichens herbarium at the Botanical Institute of Barcelona. This instance demonstrates the flexibility of the CBP to address a wide variety of needs within the biodiversity genomics community, not necessarily restricted to EBP affiliated projects.

### CBP instance

A dedicated BGP’s instance designed to support the research objectives of the CBP can be found at: https://dades.biogenoma.cat/. The CBP has as a primary aim to sequence the genomes of the eukaryotic species living in the Catalan linguistic area. The instance tracks all the data published to INSDC under the CBP umbrella (PRJEB49670), and contains, in addition, manually curated data, such as photos, vernacular names, and related publications of the sequenced organisms. This instance also provides the GoaT sequencing status report of the CBP to GoaT, thus enabling real-time updates of the CBP progress to the EBP, prior to INSDC submission.

As of February, the CBP instance contains data regarding a total of 98 organisms (Supplementary Fig. S5), 12 of them with already published assemblies [15–19].

One of the unique features tailored for this instance is the integration of region-specific taxonomic information, focusing on the biodiversity found in Catalonia. This customized taxonomic framework allows researchers to efficiently navigate and access relevant data on the local fauna and flora, facilitating more targeted research efforts. This use case illustrates the ability of the web portal to address region-specific research needs and its utility to foster collaboration within local scientific communities.

### EBP and ERGA instances

We implemented dedicated BGP instances for the EBP (https://genome.crg.es/ebp Supplementary Fig. S6) and ERGA (https://genome.crg.es/erga). These portals are designed to track all data published to the International Nucleotide Sequence Database Collaboration (INSDC) under the EBP and ERGA umbrellas.

Both portals are open to users who wish to contribute additional links and relevant data not present in INSDC for the species sequenced as part of these initiatives. The EBP and ERGA instances share some common features, such as a choropleth map (Supplementary Fig. S1) that visualize species distributions within country boundaries and the retrieval of data submitted under their respective bioproject accessions (PRJNA533106 for EBP and PRBJEB43510 for ERGA). The portals also track biosamples not yet officially associated with EBP or ERGA, including public biosamples whose metadata includes relevant project names such as “CBP,” “VGP,” or “DTOL.”

### Lichenoteca Instance

To further demonstrate the configuration flexibility of the BGP, we have also created an instance (https://genome.crg.es/lichenoteca), corresponding to a dataset from the Global Biodiversity Information Facility related to a significant lichen collection. This dataset consists of ∼5000 samples at the Institut Botànic de Barcelona [20].

The collection includes specimens gathered across Europe and North Africa from the mid-19th century to the present day. This dataset integration into the BGP not only underscores the portal’s capacity to handle diverse and complex collections, but also its adaptability in supporting a wide range of biodiversity data, from genomic sequences to dedicated specimen collections.

## Discussion

The BGP has been initially developed to meet the needs of the CBP, and the status of this EBP affiliated project can be accessed through an instance of the portal (https://dades.biogenoma.cat/.) The BGP–CBP portal is helping to coordinate the sequencing efforts within the CBP and facilitates data and metadata deposition in the designated international data resources. We believe that the portal will play a role in catalyzing collaborative projects between different research institutions affiliated to the CBP, and to extend the impact of the project beyond academic circles, engaging a wider community in biodiversity conservation and research initiatives.

A genuine focus of the bioinformatics developments within the CBP is in equity. In the framework of biodiversity genomics projects, equity is often understood as empowering researchers worldwide to produce data within the natural geographical region where the samples have been acquired. While the need for standardized analysis (mostly, gene annotations) across the tree of life may argue for centralized resources, bioinformatic pipelines do not need to be necessarily attached to specific sites. Developments in containerization software makes it possible to implement analysis pipelines producing highly reproducible results. These would contribute to democratization not only of data production, but also of data analysis [21].

The CBP will thus proactive engage in promoting the development, implementation, and use of lightweigth, autonomous, and reproducible software. We believe that the BGP is an example of such software. It does not aim to compete with the designated EBP portal (https://www.ebi.ac.uk/biodiversity/), the NCBI genome data viewer (https://www.ncbi.nlm.nih.gov/gdv) or GoaT. It rather aims to complement these efforts. It is a generic framework designed to meet the needs of small to medium-sized regional or national biodiversity genomics projects. It can be installed and run at any site. Sites could be other EBP nodes, but also countries, regions, shires, cities, neighborhoods, zoological parks, botanical gardens, museums, taxonomic groups, etc.

One of the key distinguishing features of our portal is its configuration flexibility. This flexibility allows users to integrate official metadata, such as data from INSDC, with project-defined metadata, including vernacular names, images, species geographical distributions, days of sightings, etc. In addition, the portal’s features, such as genome annotation upload and genome browser functionality, allow users to efficiently organize their data directly within the portal, streamlining data management processes.

Consequently, the portal serves multiple purposes, catering to a diverse range of users and use cases within the biodiversity field. Whether for local purposes, such as within a laboratory, botanical garden, or zoo, where only local metadata is relevant, or for tracking data exclusively published in INSDC under a Bioproject accession, or for generating GoaT reports and providing a controlled mechanism for updating the sequencing status of species, the portal’s adaptability makes it a versatile tool. Furthermore, with the appropriate customization, the portal can be used as an outreach tool helping to engage the society in the understanding of biodiversity, and, in particular, of the link between genomics and biodiversity. Genomes underline the unity of life. Understanding this promotes the perception that biodiversity is not something separate from humans, but that humans are an inextricable part of biodiversity, and, therefore, that threats to biodiversity are threats to human life on Earth.

## Supplementary Material

lqaf020_Supplemental_File

## Data Availability

A detailed description on how to configure, run, and deploy a BGP instance can be found in the README file of the github repository (https://github.com/guigolab/biogenome-portal, DOI: 10.5281/zenodo.8314305). A Catalan version of the abstract of the article can be found at: https://doi.org/10.5281/zenodo.14060240.

## Supplementary data

Supplementary data is available at NAR Genomics & Bioinformatics online.
