# Supplementary material for: The BioGenome Portal: a web-based platform for biodiversity genomics data management
Source: NAR Genom Bioinform. 2025 Mar 22;7(1):lqaf020. doi: 10.1093/nargab/lqaf020 (PMC11928930; doi:10.1093/nargab/lqaf020)
Supplement: lqaf020_Supplemental_File [file lqaf020_supplemental_file.pdf]

SUPPLEMENTARY FIGURES

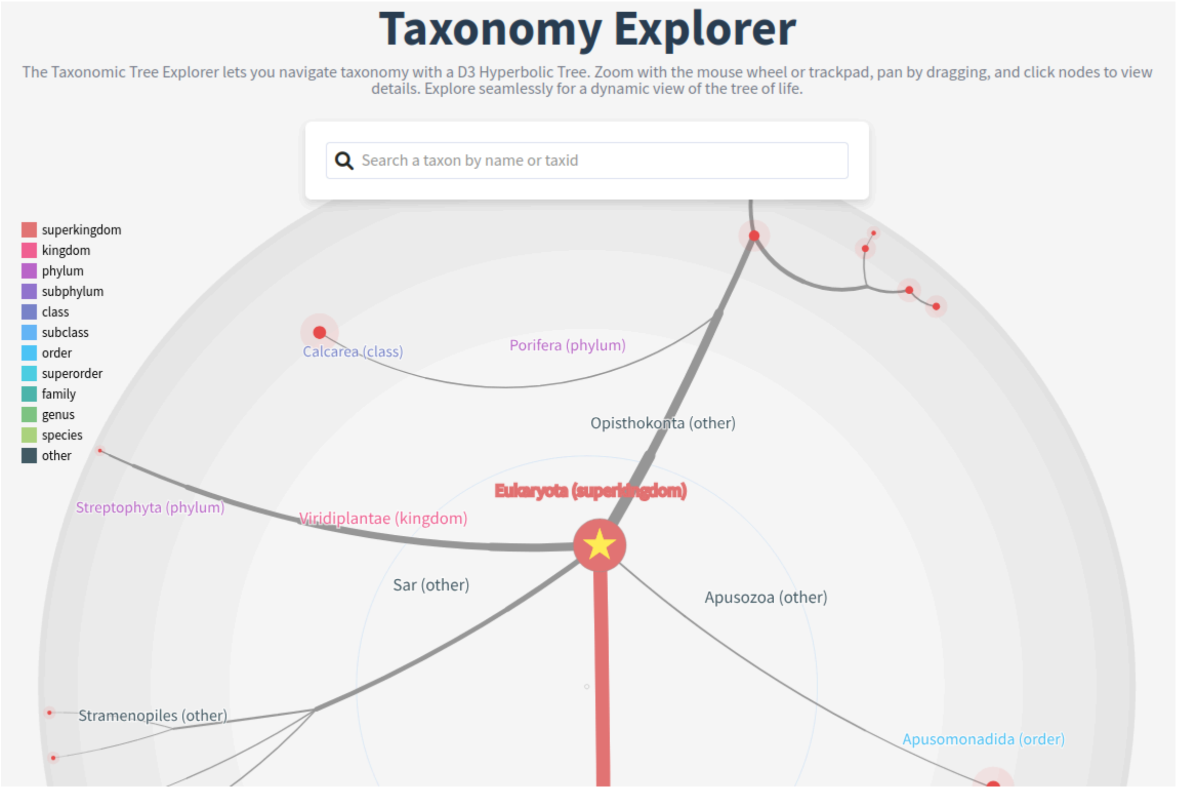

Figure S1. Taxonomy explorer (hyperbolic tree) of the CBP instance

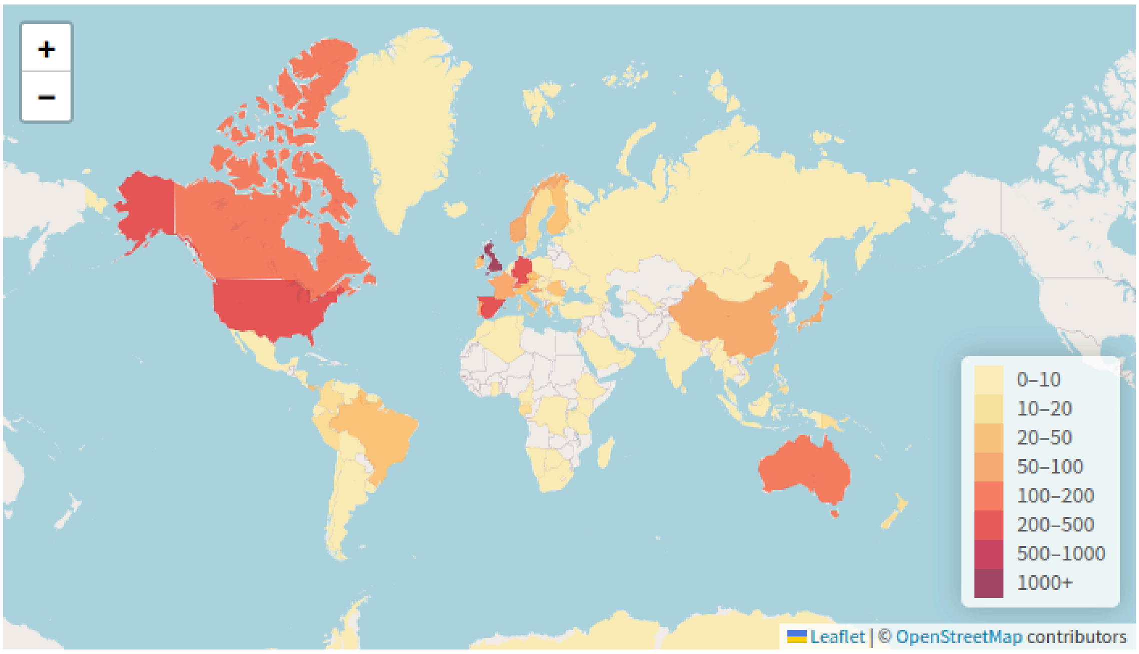

Figure S2. Choropleth map showing the distribution of organisms collected within the boundaries of each country, as seen in the organisms page of the EBP instance.

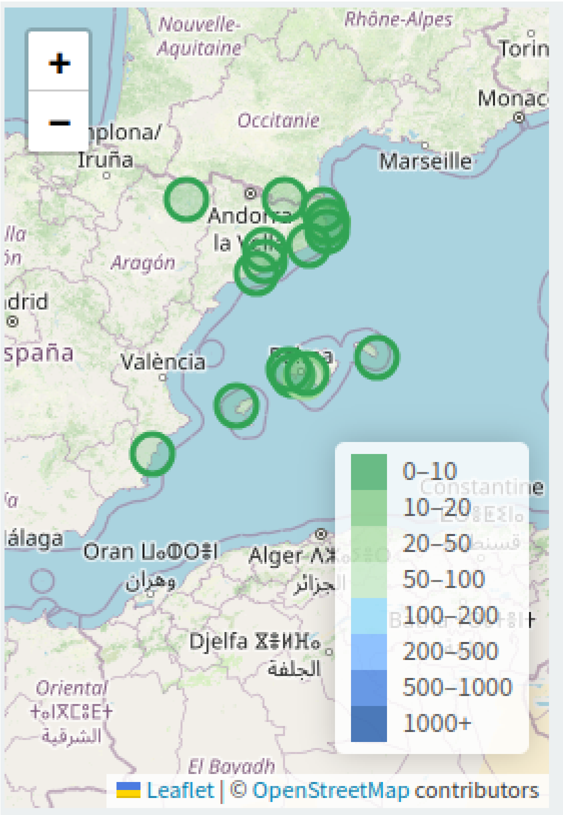

Figure S3. Geolocalization of some of the samples collected by the CBP as seen through the CBP instance.

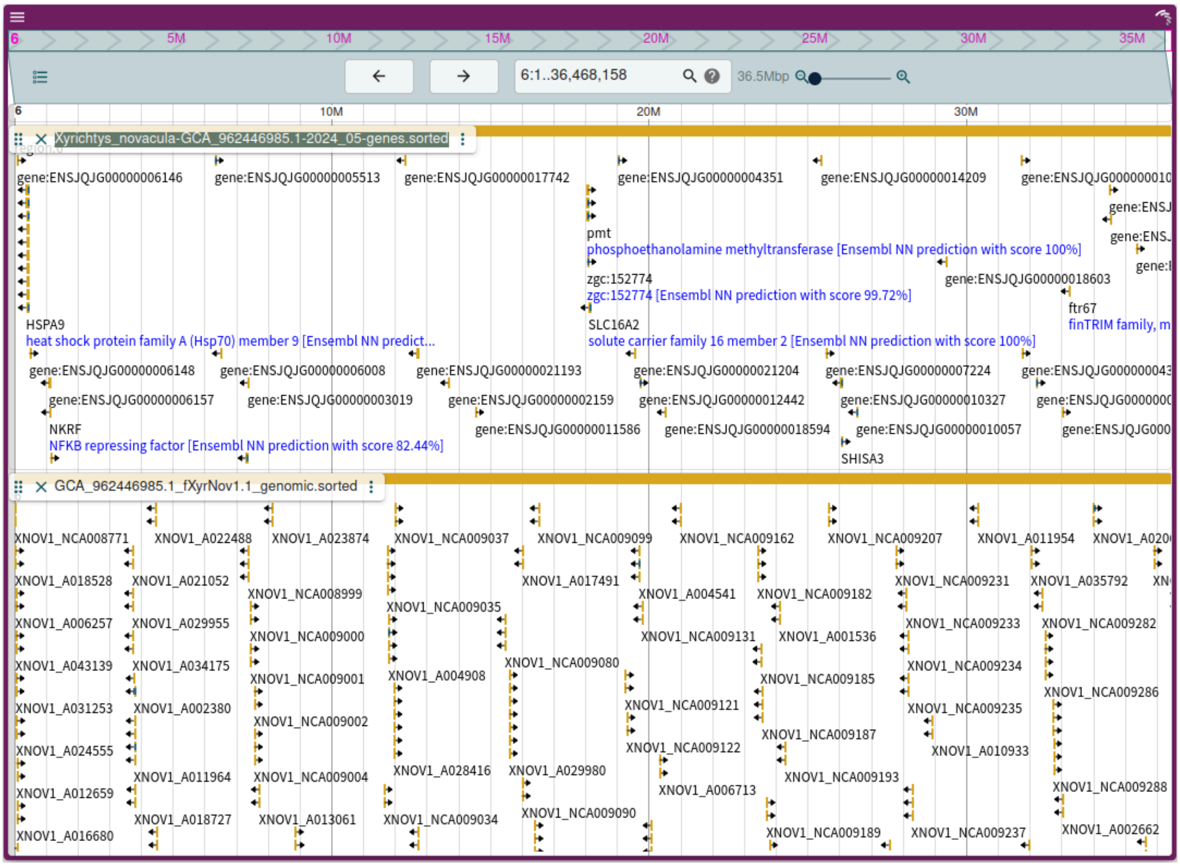

Figure S4. NCBI and Ensembl gene annotations of *Xyrichtys novacula*, a species sequenced by the CBP, as seen through the genome browser of the CBP instance

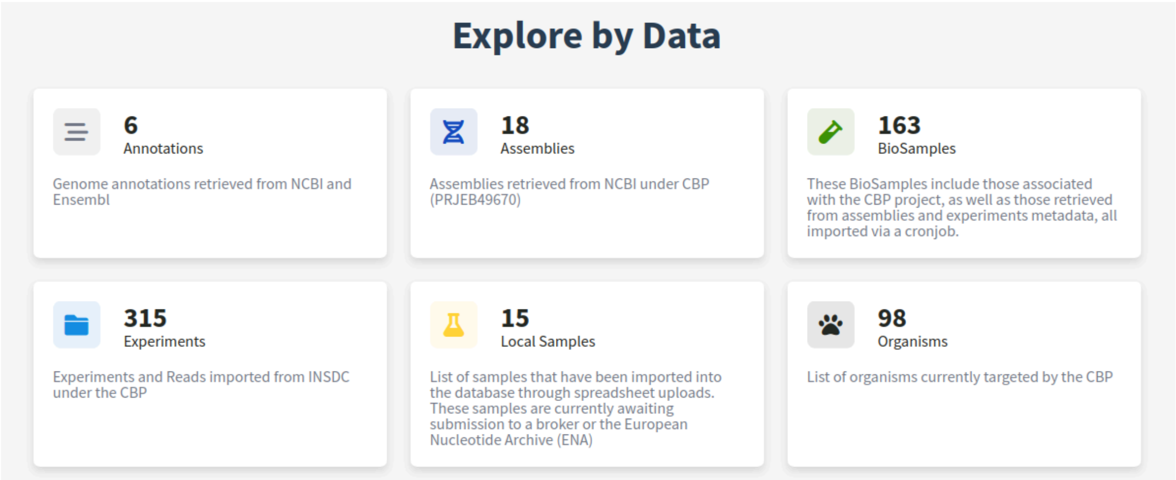

Figure S5. Data contained in the CBP instance, as of February 2025

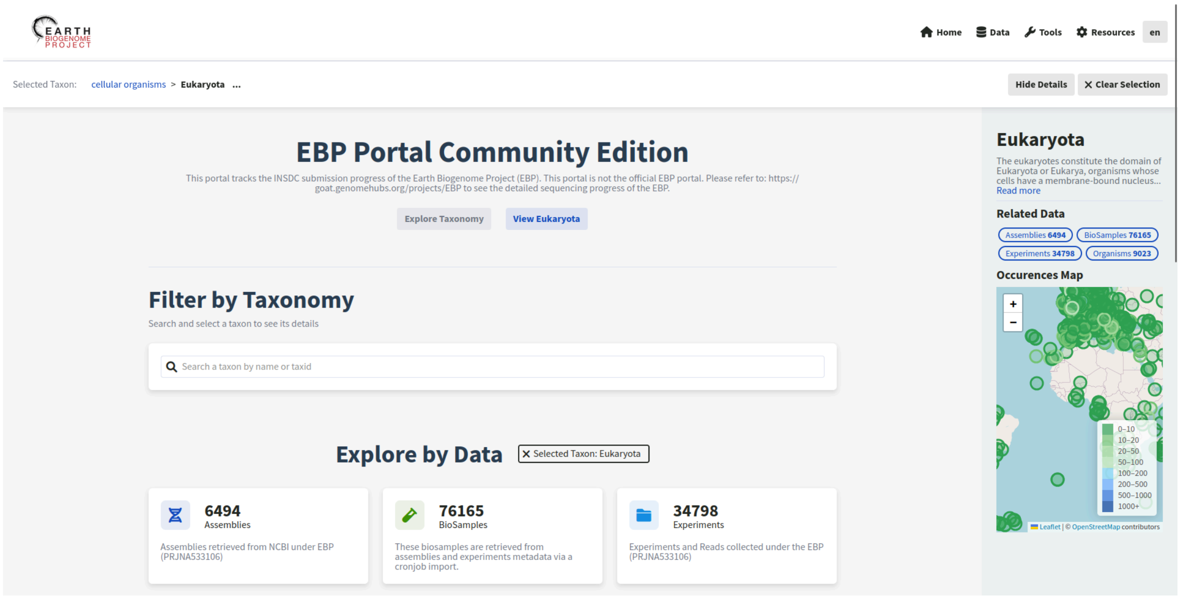

Figure S6. Landing page of the EBP instance, with the right sidebar presenting metadata for the selected taxon (Eukaryota), including the number of genome assemblies, BioSamples, experiments, and organisms and a geographic occurrences map with the sample distribution.
